# Chitosan–cellulose hydrogels: advances in stimuli-responsive biomedical therapeutics

**DOI:** 10.1039/d6ra00743k

**Published:** 2026-04-21

**Authors:** Nesa Rafati, Atefeh Zarepour, Arezoo Khosravi, Siavash Iravani, Ali Zarrabi

**Affiliations:** a Department of Biotechnology, Faculty of Biological Sciences, Alzahra University Tehran, 1953833511 Iran; b Department of Biology, Faculty of Arts and Sciences, Kocaeli University Izmit Kocaeli 41001 Türkiye; c Department of Genetics and Bioengineering, Faculty of Engineering and Natural Sciences, Istanbul Okan University Istanbul 34959 Türkiye; d Graduate School of Biotechnology and Bioengineering, Yuan Ze University Taoyuan 320315 Taiwan; e Independent Researcher W Nazar ST, Boostan Ave Isfahan Iran siavashira@gmail.com; f Department of Biomedical Engineering, Faculty of Engineering and Natural Sciences, Istinye University Istanbul 34396 Türkiye alizarrabi@gmail.com

## Abstract

Chitosan–cellulose hydrogels have emerged as versatile stimuli-responsive biomaterials for biomedical therapeutics, combining the antimicrobial, mucoadhesive, and pH-sensitive properties of chitosan with the mechanical strength, high water retention, and biodegradability of cellulose. These hybrid systems have demonstrated significant potential in controlled drug delivery, tissue engineering, and regenerative medicine, enabling sustained and localized therapeutic release while reducing systemic toxicity in cancer and chronic disease management. Despite these advantages, important challenges remain regarding long-term *in vivo* biocompatibility, immune responses, and the biological fate of degradation products, which may influence systemic inflammation and organ function. Furthermore, precise control over multi-stimuli responsiveness, mechanical stability, and degradation kinetics remains critical for clinical translation. Recent advances in nanocomposite strategies, including integration with graphene-based materials, magnetic nanoparticles, and other functional nanomaterials, have introduced next-generation hybrid platforms with enhanced mechanical, electrical, and targeted therapeutic capabilities. Although several preclinical studies have demonstrated promising outcomes, further systematic *in vivo* investigations and translational research are required to ensure safety, reproducibility, and clinical applicability. This review provides a comprehensive overview of recent advances in chitosan–cellulose hydrogels, critically discusses current limitations, and highlights emerging hybrid systems and future directions toward next-generation biomedical applications.

## Abstract 2

Chitosan–cellulose hydrogels are versatile stimuli-responsive biomaterials for therapeutics, combining chitosan's antimicrobial, mucoadhesive, pH-sensitive properties with cellulose's strength, water retention, and biodegradability.

## Graphical abstract

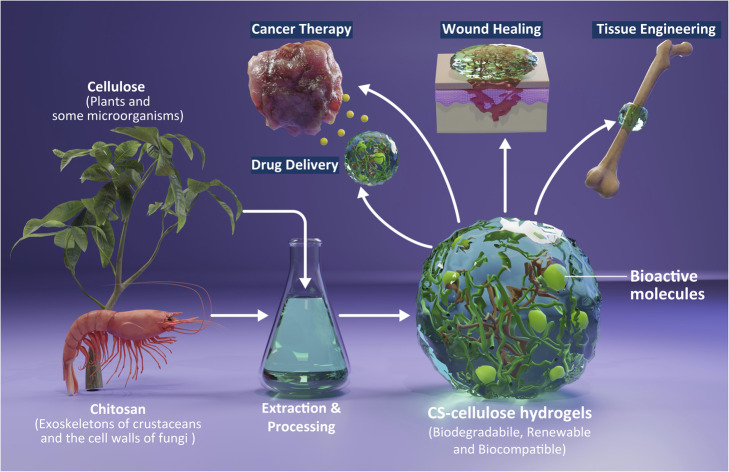

## 1 Introduction

Natural polymers have attracted significant attention in recent decades as sustainable alternatives to synthetic materials.^1^ Cellulose and chitosan (CS) are among the most usable biopolymers due to their interesting properties including their abundance, renewability, and excellent biocompatibility. These are polysaccharides polymers with different structural similarities which are derived from distinct natural sources and possess different physicochemical and functional properties. The complementary features of these biopolymers make them highly promising candidates for the development of advanced multifunctional materials.^2^ Cellulose is the most abundant natural biopolymer on Earth and is primarily derived from plants, where it functions as the main structural component of plant cell walls. Structurally, cellulose is a linear polysaccharide composed of repeating β-(1 → 4)-linked d-glucose units that assemble into long chains stabilized by extensive intra- and intermolecular hydrogen bonding. This highly organized hydrogen-bonding network confers remarkable crystallinity, mechanical strength, and chemical resistance to cellulose.^3^ In nature, cellulose predominantly exists as native cellulose (cellulose I), which is found in plant cell walls and bacterial biofilms, whereas regenerated cellulose (cellulose II) is obtained through chemical or physical processing and typically exhibits higher chemical reactivity.^4^ The presence of hydroxyl groups at the C-2, C-3, and C-6 positions of each anhydroglucose unit provides multiple reactive sites for chemical modification. These functional groups enable the synthesis of a wide range of cellulose derivatives, such as cellulose acetate, carboxymethyl cellulose (CMC), hydroxyethyl cellulose (HEC), and methyl cellulose (MC), which improve the solubility, flexibility, and compatibility of cellulose with other polymers.^5^ As a result, cellulose-based materials exhibit several advantageous properties, including mechanical robustness, biocompatibility, biodegradability, hydrophilicity, thermal and chemical stability, and excellent surface modifiability.^4^ In recent years, the emergence of nanocellulose, including cellulose nanocrystals (CNCs) and cellulose nanofibers (CNFs), has further expanded the potential of cellulose-based materials. Owing to their high surface area, tunable crystallinity, and outstanding reinforcing capability, these nanostructures have attracted considerable attention for the fabrication of advanced nanocomposites with enhanced mechanical and functional performance.^6^

CS is the second most abundant natural polymer which is derived from chitin, a structural polysaccharide commonly found in the exoskeletons of crustaceans and the cell walls of fungi.^7^ It is a linear cationic polysaccharide consists of β-(1 → 4)-linked d-glucosamine and *N*-acetyl-d-glucosamine units which is produced through the partial deacetylation of chitin. The degree of deacetylation plays a critical role in determining its physicochemical characteristics, including solubility, charge density, and biological activity.^8^ The presence of primary amino groups (–NH_2_) at the C-2 position of glucosamine units enables protonation under acidic conditions, providing a positive charge to CS and facilitating electrostatic interactions with negatively charged biomolecules such as proteins, DNA, and microbial cell surfaces.^9^ This intrinsic cationic nature differenciates CS from most other naturally occurring polymers, which are typically neutral or anionic. In addition to its cationic character, the abundance of amino and hydroxyl functional groups provides multiple reactive sites for chemical modification, including acylation, phosphorylation, carboxymethylation, and quaternization. Such derivatization strategies significantly enhance the solubility, stability, and bioavailability of CS, thereby expanding its functional versatility.^10^ Therefore, CS exhibits a broad spectrum of desirable physicochemical and biological properties, including biocompatibility, biodegradability, antimicrobial activity, film- and hydrogel-forming capability, mucoadhesiveness, and hemostatic activity. The antimicrobial activity of CS is primarily attributed to electrostatic interactions between its positively charged groups and negatively charged microbial cell membranes, which can disrupt membrane integrity and lead to the leakage of intracellular components.^11^ Furthermore, its hemostatic and adhesive properties allow efficient interaction with biological tissues and promote blood clot formation.^11,12^

Owing to these unique characteristics, CS has been extensively investigated in a wide range of biomedical and environmental applications, including drug delivery systems, wound healing materials, tissue engineering scaffolds, and biosorbents for wastewater treatment. Moreover, its ability to interact with both organic and inorganic components makes CS an attractive platform for the fabrication of multifunctional composite materials with tailored physicochemical and biological functionalities.^8,13,14^

The integration of cellulose and CS creates a synergistic platform that combines the mechanical strength of cellulose with the intrinsic bioactivity of CS, resulting in composites with enhanced physicochemical and biological performance.^2^ These two biopolymers interact primarily through hydrogen bonding between the hydroxyl groups of cellulose and the amino or hydroxyl groups of CS, as well as electrostatic interactions under acidic conditions. Such intermolecular interactions promote strong interfacial adhesion, improved structural stability, and tunable porosity within the resulting composite materials.^15^ While cellulose contributes structural integrity, dimensional stability, and mechanical strength, CS provides antimicrobial, antioxidant, and hemostatic functionalities. This complementary relationship makes cellulose–CS composites particularly attractive for applications that require both mechanical durability and biological activity.^16^

Cellulose–CS composites can be fabricated into a variety of structural architectures, including hydrogels and aerogels, films and membranes, fibers and nanofibers, sponges and foams, as well as functional coating.^17–21^ Owing to their unique structural and functional characteristics, cellulose–CS composites have demonstrated significant potential across a wide range of biomedical applications. For example, in wound healing, composite hydrogels and films can maintain a moist healing environment, promote cell adhesion, prevent microbial infection, and accelerate tissue regeneration. The combination of cellulose's mechanical strength with the antimicrobial activity of CS leads to highly efficient wound dressing materials.^22^ In addition, these composites can be engineered to exhibit stimuli-responsive behavior, such as pH- or redox-triggered responses, enabling controlled drug release of therapeutic agents including antibiotics or natural compounds such as curcumin.^23^ Furthermore, porous cellulose–CS scaffolds can mimic the extracellular matrix (ECM), supporting fibroblast proliferation and tissue regeneration, which is essential for tissue engineering applications.^24^ Therefore, the integration of cellulose and CS represents a powerful approach to designing multifunctional, sustainable materials for biomedical applications.

Among different formulation, chitosan–cellulose hydrogels have emerged as a promising class of biopolymer-based materials due to the synergistic combination of the structural rigidity of cellulose and the bioactivity of chitosan. These hydrogels are typically formed through physical (hydrogen bonding) and/or chemical crosslinking between the hydroxyl groups of cellulose and the amino and hydroxyl groups of chitosan, resulting in three-dimensional porous networks with tunable physicochemical properties. The presence of abundant reactive functional groups enables the formation of stable hydrogel matrices with enhanced mechanical strength, swelling capacity, and controlled degradation behavior.^25,26^ In here, the degree of deacetylation of chitosan is a key structural parameter that critically controls its interaction with cellulose and the formation of hydrogel networks. As mentioned earlier, the degree of deacetylation reflects the proportion of deacetylated glucosamine units bearing free amino groups (–NH_2_), which directly determine the charge density, solubility, and reactivity of chitosan. Recent studies have demonstrated that increasing the degree of deacetylation (typically in the range of ∼80–95%) enhances the protonation of amino groups under acidic conditions, thereby strengthening electrostatic interactions with negatively charged cellulose derivatives.^27,28^ This increased cationic character promotes the formation of stable polyelectrolyte complexes and facilitates hydrogel network formation through combined ionic interactions and hydrogen bonding. In particular, it was shown that variations in the degree of deacetylation significantly affect the formation and stability of chitosan–cellulose complexes, where higher degree of deacetylation leads to stronger intermolecular associations and improved network integrity.^28^ Moreover, the degree of deacetylation influences key hydrogel properties including swelling behavior, mechanical strength, and degradation rate, which are essential for biomedical performance. For example, higher degree of deacetylation of chitosan generally yields hydrogels with enhanced structural stability and more controlled swelling profiles due to increased crosslinking density.^29^ Therefore, precise control of chitosan the degree of deacetylation is essential for modifying the physicochemical properties and functional performance of cellulose–CS hydrogels in advanced biomedical systems.

**Fig. 1:**
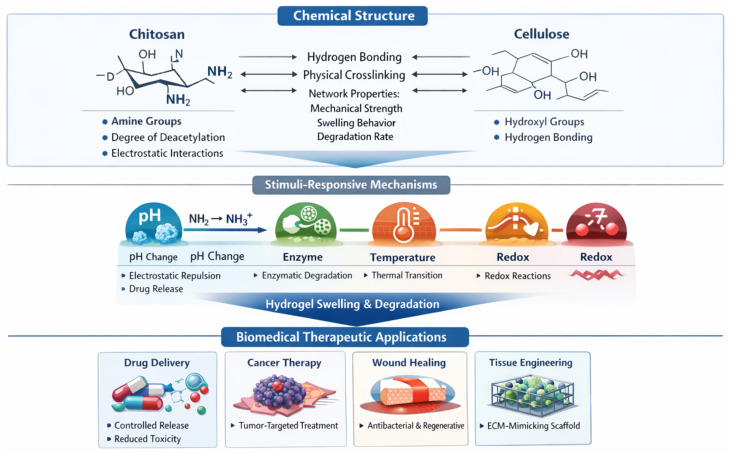
Structure–activity relationships controlling the biomedical performance of CS–cellulose hydrogels. The functional groups of CS (amine groups) and cellulose (hydroxyl groups) regulate intermolecular interactions such as hydrogen bonding and electrostatic forces, which determine crosslinking density, swelling behavior, and degradation kinetics. External stimuli including pH, enzymes, temperature, and redox conditions modulate hydrogel responsiveness, enabling controlled drug release and adaptive biological interactions. These structure-dependent properties enable diverse biomedical applications including drug delivery, cancer therapy, wound healing, and tissue engineering.

Stimuli-responsive chitosan–cellulose hydrogels represent an advanced class of smart biomaterials capable of dynamically responding to external or internal environmental cues such as pH, temperature, light, ionic strength, and biochemical signals. These systems use the intrinsic pH-sensitive behavior of chitosan, deriving from the protonation/deprotonation of amino groups, in combination with the structural stability and hydrophilicity of cellulose to form tunable hydrogel networks. Recent studies have shown that incorporating functionalized cellulose derivatives (*e.g.*, hydroxyethyl cellulose or nanocellulose) into chitosan matrices enhances responsiveness, enabling controlled swelling, degradation, and drug release in response to physiological triggers.^30,31^ In addition, the integration of nanomaterials or functional moieties can introduce multi-stimuli responsiveness, including thermo-, photo-, and electro-responsive behaviors, further broadening their biomedical applicability. These smart hydrogels demonstrated significant potential in drug delivery, wound healing, and tissue engineering, where their ability to modulate physicochemical properties *in situ* enables precise control over therapeutic release profiles and cellular interactions. Therefore, the synergistic combination of chitosan and cellulose provides a versatile platform for designing next-generation stimuli-responsive hydrogels with enhanced functionality and adaptability for personalized biomedical applications.^30,32,33^ The structure-activity relationships controlling the stimuli-responsive behavior and biomedical performance of chitosan–cellulose hydrogels are illustrated in Fig. 1.

**Fig. 2:**
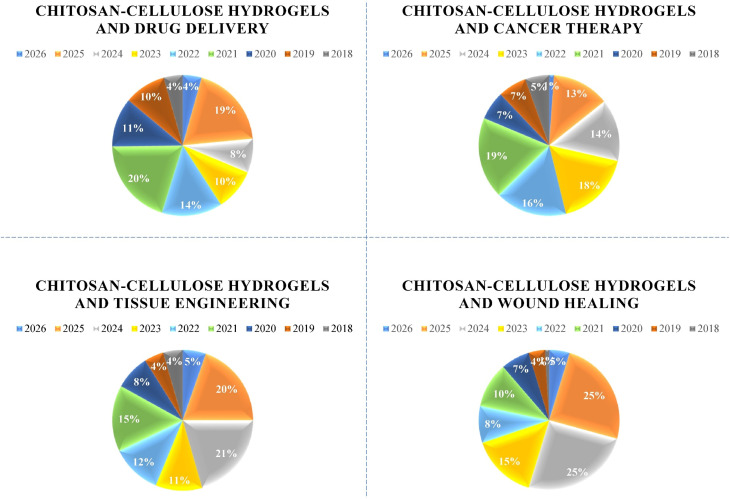
Web of science data about the number of publications using different keywords including “chitosan–cellulose hydrogels and drug delivery”, “chitosan–cellulose hydrogels and cancer Therapy”, “chitosan–cellulose hydrogels and tissue engineering”, and “chitosan–cellulose hydrogels and wound healing”.

This review aimes to highlight recent biomedical advances in CS–cellulose hydrogels, with particular emphasis on their emerging roles in tissue engineering and regeneration, wound healing, and controlled drug delivery. To this aim, we have checked and explained researches done in last nine years (Fig. 2). By examining recent progress in fabrication strategies, chemical modifications, and stimuli-responsive designs, this review aims to explain how these developments enhance the functional performance of CS–cellulose hydrogels for biomedical applications. Furthermore, it identifies current limitations and research gaps while providing perspectives on future directions that may facilitate the clinical translation of these biomaterials into effective regenerative therapies. Overall, this review highlights the transformative potential of CS–cellulose hydrogels as versatile and sustainable platforms capable of addressing critical biomedical challenges through tailored and multifunctional solutions.

## 2 Biomedical application of chitosan–cellulose hydrogels

### 2.1 Drug delivery and cancer therapy

Cellulose–CS hydrogels have emerged as promising platforms for targeted drug delivery due to their remarkable biocompatibility, biodegradability, and tunable physicochemical properties. These hydrogels can enhance the circulation time of loaded drugs by providing a protective matrix that reduces early breakdown and removal.^34,35^ The incorporation of cellulose nanocrystals into the CS matrix significantly enhances mechanical stability through strong polymer–nanocrystal interactions, effectively overcoming the intrinsic weakness of pure chitosan hydrogels.^36^ The resulting composite networks typically exhibited a highly porous structure that facilitated drug encapsulation and controlled diffusion, with release kinetics tunabled by varying CNC content.^37^ Beyond their role as drug carriers, CS–CNC hydrogels provided a favorable microenvironment for cell adhesion and osteogenic differentiation, promoting mineral deposition and upregulation of osteogenic markers in bone-related applications.^38^ Their inherent antibacterial activity further expands their potential in regenerative medicine by reducing infection risk at the implant site.^39^ These CS–CNC hydrogel scaffolds demonstrated a versatile class of polysaccharide-based biomaterials that combine mechanical robustness, biocompatibility, and controlled release behavior for advanced tissue engineering and therapeutic delivery systems. In this context, a dual-network hydrogel strategy was introduced to overcome the intrinsic trade-off between pH sensitivity and mechanical stability inherent to hydrogel-based delivery systems. In this design, the integration of CMC/CS and sodium alginate/calcium chloride (SA/Ca^2+^) networks produced hydrogel beads featuring a reticulated shell structure capable of maintaining mechanical integrity under acidic conditions, while responding selectively to intestinal pH. This design maintained mechanical integrity under acidic conditions while allowing selective responsiveness to intestinal pH. Notably, the CMC/CS/SA bead demonstrated superior pH responsiveness, characterized by the most pronounced increase in swelling behavior and a maximum swelling ratio of 22%. The encapsulation efficiency of *Bacillus subtilis natto* reached 67.3%, and the formulation enabled sustained release for over 10 h. As a result, the beads remained stable in the gastric environment but gradually disintegrated in the intestines, enabling controlled, site-specific release of encapsulated probiotics, such as *Bacillus subtilis natto*. The dual-network architecture achieved a favorable balance between robustness and responsiveness, supporting sustained release and prolonged microbial viability. This strategy demonstrated how rational polymer design can harmonize environmental sensitivity with long-term stability, providing a versatile framework for advanced hydrogel formulations in both biomedical and food delivery applications.^40^

**Fig. 3:**
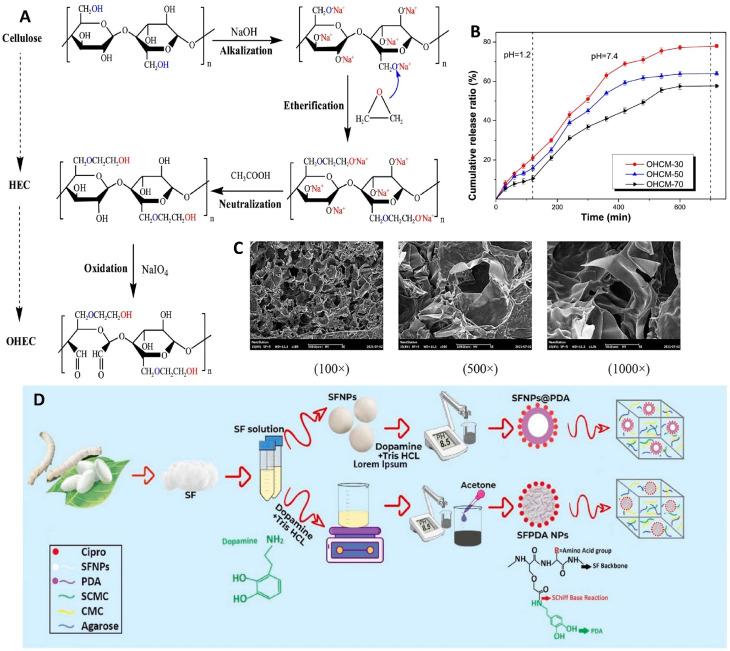
(A) Oxidation process of pineapple peel cellulose. (B) The release profile of bovin serum albumin from different OHCM hydrogels (with different oxidation degree) at different pH. (C) Scanning electron microscopy (SEM) images of the OHEC-70/CMCS hydrogel at different magnifications. Reprinted with permission from ref. 41. Copyright 2021, American Chemical Society. (D) Simple illustration of preparing the CMC/SCMC/agarose hydrogel and adding ciprofloxacin-loaded SF nanoparticles with and without PDA, showing their pH-responsive drug release. Reprinted with permission from ref. 42. Copyright 2022, Elsevier B.V. (B).

The integration of oxidized hydroxyethyl cellulose derived from pineapple peel with carboxymethyl chitosan obtained from *Hericium erinaceus* residue led to the production of a smart pH-responsive hydrogel system *via* fabricating a sustainable and biodegradable polymeric network through dynamic Schiff-base interactions (Fig. 3A–C). The hydrogel exhibited a porous three-dimensional structure with interconnected networks, which facilitated efficient water uptake and drug diffusion. Swelling studies demonstrated a clear pH-dependent behavior, with significantly higher swelling ratios under neutral physiological pH (7.4) compared with acidic and basic pH. *In vitro* release experiments revealed a controlled and sustained release profile, with cumulative drug release reaching approximately 80% under normal conditions within 700 min, compared with slower release (∼30%) at pH 1.2, confirming its pH-sensitive release behavior, appropriate it for gastrointestinal applications.^41^

A multifunctional hydrogel dressing was developed by combining CMCS, CMC, and agarose, incorporating silk fibroin (SF)/polydopamine nanoparticles as a drug delivery component (Fig. 3D). The formation of a stable three-dimensional network was confirmed through physicochemical characterization, revealing a highly porous and interconnected structure that facilitated fluid absorption and drug diffusion. The introduction of nanoparticles enhanced the mechanical strength and structural stability of the hydrogel, with compressive strength and elasticity showing noticeable improvement compared to the nanoparticle-free system. Swelling studies demonstrated a high water absorption capacity, with equilibrium swelling ratios typically reaching approximately 1000–1600%, indicating suitability for wound exudate management. The hydrogel exhibited pH-responsive behavior, where increased swelling and drug diffusion were observed under slightly acidic conditions relevant to infected wound environments. Drug loading efficiency was reported to be high, generally in the range of ∼80.74%, due to strong interactions between the drug molecules and the functional groups within both the polymeric matrix and the nanoparticles. *In vitro* drug release experiments showed a sustained and controlled release profile, with cumulative release reaching approximately 80% over 21 days at basic pH compared with about 50% and 55% under acidic and neutral pH. The presence of polydopamine contributed to improved drug retention and prolonged release behavior. Antibacterial evaluation demonstrated high efficacy, with inhibition rates exceeding ∼95% against *Pseudomonas aeruginosa* (*P. aeruginosa*) with better effect at alkalin condition. Besides, more than 80% antibacterial activity were observed after 15 days incubation, confirmed the effectiveness of fabricated formulation.^42^

Cellulose–CS hydrogels have also attracted considerable attention as advanced platforms for cancer therapy, primarily due to their excellent biocompatibility, biodegradability, and highly tunable physicochemical properties. These hydrogel systems can improve the pharmacokinetic profile of anticancer agents by providing a protective three-dimensional matrix, thereby reducing premature degradation and rapid systemic clearance. In addition, their inherent mucoadhesive characteristics and pH-responsive behavior enable site-specific and controlled drug release, particularly within the mildly acidic tumor microenvironment, which contributes to enhanced therapeutic efficacy while minimizing off-target toxicity.^34,35^ They can be engineered to interact with the tumor microenvironment, which is often leaky and more permeable to macromolecules, facilitating enhanced drug accumulation at the target site. Therefore, these biopolymer-based hydrogels introduce an advanced approach for polymeric-nanodrug conjugates, offering controlled release, improved bioavailability, and synergistic therapeutic actions in precision medicine applications.^43,44^ Recent advances in polysaccharide-based nanocarriers have opened promising ways for targeted cancer therapy. For instace, the advancement in dynamic polysaccharide-based materials have led to the development of a novel class of pH-responsive, injectable, and self-healing hydrogels formed through Schiff base reactions between oxidized hydroxypropyl cellulose (Ox-HPC) and carboxymethyl chitosan (CMCS). The interaction between carbonyl groups of Ox-HPC and amino groups of CMCS in aqueous media produced a dynamic network stabilized by reversible imine bonds. This reversible crosslinking provided self-healing ability, injectability, and adaptability, making the hydrogels suitable for localized and minimally invasive biomedical applications. Structural and rheological analyses confirmed the formation of a stable, porous three-dimensional network at optimized compositions (20% Ox-HPC and 5% CMCS). The hydrogel with the lowest crosslinking, swelled rapidly and reached a swelling ratio of approximately 12.3 g g^−1^ in water. The hydrogels exhibited excellent mechanical resilience and rapid recovery after deformation, validating the dynamic reversibility of the imine bonds. Furthermore, oxidation and neutralization treatments enhanced Ox-HPC solubility and uniformity, ensuring reproducible hydrogel formation. The fabricated hydrogel showed pH-dependent drug release behavier, with significantly faster release at pH 6.8 (representing the tumor microenvironment) compare with physiological pH. This tunable responsiveness highlighted the potential of this material for site-specific delivery in acidic pathological conditions, such as tumor tissues. The biocompatibility, injectability, self-repairing behavior, and environmentally safe synthesis made this hydrogel a promising platform for localized tumor therapy, tissue regeneration, and controlled drug delivery.^45^

**Fig. 4:**
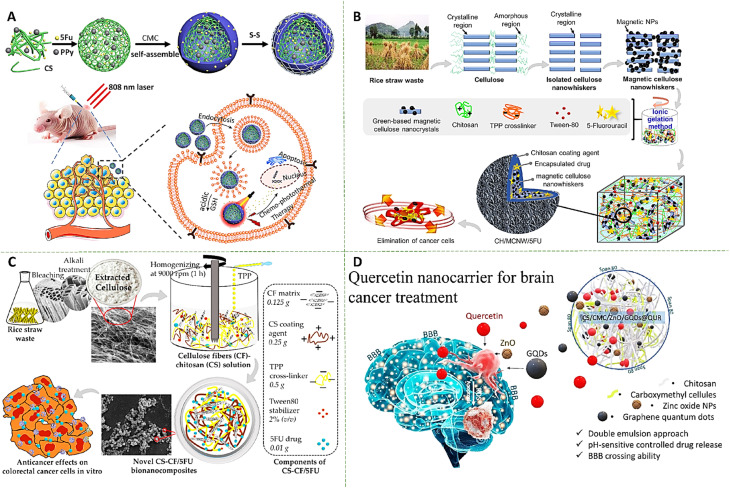
(A) The preparation of drug-loaded CS NPs and CMC/CS NP composites, along with the evaluation of their therapeutic effects in a mouse model. Reprinted with permission from ref. 46. Copyright 2021, Elsevier Ltd. (B) Schematic illustration of the CH/MCNW/5FU nanocomposite system, showing chitosan–coated magnetic cellulose nanowhiskers as the drug carrier, 5-fluorouracil loading, and its potential for enhanced anticancer activity and tumor penetration in colorectal cancer models. Reprinted with permission from ref. 47. Copyright 2023, Elsevier B.V. (C) CS–CF/5FU bio-nanocomposites were engineered by encapsulating 5-fluorouracil within a chitosan–cellulose fiber matrix, yielding a stable and biocompatible nanoformulation. This system was subsequently employed for comprehensive *in vitro* cytotoxicity evaluations against colorectal cancer cell lines. Reprinted from ref. 48 under the terms and conditions of the Creative Commons Attribution (CC BY) license. Copyright 2021, MDPI. (D) The CS/CMC/GQDs/ZnO@QUR nanocomposite with nanoneedle morphology, for enhanced quercetin delivery across the blood–brain barrier, showing controlled drug release and potent anticancer activity against U-87 MG brain tumor cells while maintaining low cytotoxicity. Reprinted with permission from ref. 49. Copyright 2023, Elsevier B.V.

A redox-responsive CMC/CS complex nanoparticle system was developed to achieve synergistic chemo-photothermal treatment (Fig. 4A). The nanoparticles were fabricated through a simple electrostatic adsorption method, incorporating polypyrrole (PPy) and 5-Fu within a CS core, coated with disulfide-crosslinked CMC. This dual-layered structure provided the system with excellent responsiveness to multiple tumor-specific stimuli, including near-infrared (NIR) irradiation, elevated glutathione (GSH) levels, and weakly acidic pH. The fabricated nanocomposite hydrogel (CMC/CS@PPy loaded 5Fu) exhibited high drug encapsulation efficiency (∼74.9 ± 0.3%) with a drug loading capacity of approximately 24.2 ± 0.2%, enabling sustained drug delivery reaching nearly 75% cumulative release within 24 h under the presence of NIR irradiation, redox condition and acidix pH (6.5) compared to 30% release under normal conditions. *In vitro* studies confirmed efficient cellular uptake by HepG2 liver cancer cells, with nearly 95% cell inhibition at the concentration of 100 µg mL^−1^ in the presence of redox condition and NIR irradiation. Moreover, *in vivo* assessments on HepG2 xenograft tumors showed significant inhibition in increasing the size of tumor along with inducing necrosis in tumor cells during 21 days exposer with nanocomposite hydrogel and NIR irradiation, compared with other treatments that confirmed its anti-tumor capability.^46^

Cellulose nanowhiskers, Fe_3_O_4_ nanoparticles, and CS were used in another research to fabricate an anticancer magnetic nanocomposites loaded with FU, as an anticancer therapeutic compound (Fig. 4B). Comprehensive structural analyses confirmed the successful synthesis and uniform integration of all components. The resulting nanocomposites exhibited spherical morphology with an average diameter of ∼37.16 ± 3.08 nm, positive surface charge due to the CS coating, and favorable stability for biomedical applications. The fabricated formulation showed drug encapsulation efficiency of about 92 ± 3.58%, indicating effective interaction between the drug and the polymeric matrix. *In vitro* studies demonstrated effective cytotoxic activity against colorectal cancer cells in both 2D and 3D models, while showing relatively lower toxicity toward normal colon fibroblasts. Therefore, these findings highlighted CS-coated magnetic cellulose nanowhiskers as a promising, low-cost, and multi-functional platform for targeted drug delivery in colorectal cancer therapy.^47^

Using rice straw-derived cellulose, the spherical CS nanoparticles, and 5-FU led to the production of an engineered bionanocomposite as a multifunctional platform for colorectal cancer therapy (Fig. 4C). The incorporation of cellulose fibers within the chitosan matrix not only enhances the mechanical integrity of the system but also modulates its swelling behavior, thereby influencing drug diffusion kinetics in a biologically relevant manner. A specific feature of the formulation is its pronounced pH-responsive release profile, controlled by the protonation–deprotonation dynamics of chitosan. Under mildly acidic conditions (pH ≈ 5.5), which mimic the tumor microenvironment and endo/lysosomal compartments, the system exhibited accelerated swelling and polymer relaxation, resulting in a cumulative drug release of approximately 85% within 72 h, compared with ∼50% at normal condition, thereby demonstrating effective suppression of premature drug leakage and enhanced site-selective delivery. This responsive behavior translated into increased anticancer efficacy against colorectal cancer cell models. The fabricated bionanocomposites induced a marked, dose-dependent reduction in cell viability, achieving up to ∼56.42 ± 0.41% and 8.16 ± 2.11% inhibition at the concentration of 250 µg mL^−1^ of HCT116 cancer cell and CCD112 colon normal cell, respectively, which confirmed the high anticancer activity of the fabricated formulation.^48^

The hybrid CS/CMC polymeric network, reinforced with zinc oxide (ZnO) and graphene quantum dots (GQDs) formed a robust and highly stable matrix loaded with encapsulating quercetin (QUR) for the aim of brain cancer therapy (Fig. 4D). The strong intermolecular interactions, including hydrogen bonding between CS, CMC, and quercetin molecules, as well as interactions with ZnO and GQDs, prevented fast drug leakage and improve stability, ensuring sustained release. The fabricated system exhibited significant pH-responsive behavior, with swelling and drug release markedly enhanced under mildly acidic conditions that mimic the tumor microenvironment. The fabricated formulation showed loading efficiency and encapsulation efficiency of about 46.75% and 87.5%, respectively, which were higher than the previous works. *In vitro* release studies demonstrated a sustained and controlled release profile, with cumulative quercetin release reached to about 95% over 96 h under acidic conditions, where as a slower release (∼56%) was observed at neutral pH. *In vitro* cytotoxicity effects agains L929 normal cell showed very low toxicity effect (∼15%), while the cell viability reached to 49% in the case of U-87 glioma cancer cell, which confirmed high anticancer capability of this formulation.^49^

Smart hydrogel-based drug delivery systems capable of releasing multiple therapeutic agents in a controlled and stimulus-responsive manner are gaining increasing attention for enhanced cancer therapy. In this context, a dual-responsive nanocomposite hydrogel system integrating mesoporous silica nanoparticles (MSNs) with polysaccharide networks was developed to enable the co-delivery of hydrophilic and hydrophobic anticancer agents with high efficiency and selectivity. In this platform, aminated MSNs significantly enhanced drug loading, achieving a cytarabine (Cyt) loading efficiency of 74.9%, compared to 39.2% for non-functionalized carriers, while maintaining a uniform particle size distribution of approximately 500–600 nm. Notably, the system exhibited noticeable dual stimuli-responsiveness, enabling sequential drug release under tumor-relevant conditions. The methotrexate (MTX) release was strongly pH-dependent, reaching 79.76% at pH 5.0 within 12 h, compared to 45.65% at pH 6.8 and only 16.76% at physiological pH 7.4, due to the acid-labile cleavage of acylhydrazone bonds within the hydrogel network. In parallel, Cyt release was governed by redox sensitivity, with up to 92.24% release in the presence of 20 mM glutathione (GSH), while significantly lower release levels of 31.66% and 17.63% were observed at 5 and 1 mM GSH, respectively, confirming efficient disulfide bond cleavage under intracellular reducing conditions. *In vitro* assessments confirmed the excellent biocompatibility of drug free nanoformulation, maintaining cell viability above 95% even at high concentrations (100 µg mL^−1^), whereas the dual-drug formulation demonstrated markedly enhanced anticancer efficacy, reducing HepG2 cell viability to 10.2%, significantly outperforming single-drug systems, which retained 36.87% and 45.10% viability under identical conditions. Therefore, this study demonstrated that integrating pH- and redox-responsive mechanisms within hybrid nanostructured hydrogels enabled controlled, sequential drug delivery and synergistically improved therapeutic outcomes in cancer treatment.^50^

In another research, an advanced pH- and near-infrared (NIR)-responsive hydrogel system was developed based on oxidized carboxymethyl cellulose (oxCMC) and CS, co-doped with graphene oxide (GO), as an advanced platform for controlled delivery of 5-fluorouracil (5-FU). The hydrogel was formed *via* Schiff base crosslinking, where acid-labile –HC=N– bonds endowed the system with pH-sensitive behavior, enabling enhanced swelling and drug release under acidic conditions. Specifically, the swelling ratio increased significantly from 23.5% at pH 7.4 to 56.4% at pH 5.0, which directly facilitated drug diffusion in tumor-mimicking environments. In parallel, the incorporation of GO introduced a photothermal-responsive feature, allowing NIR-triggered hyperthermia to further accelerate drug release. As a result, the cumulative release of 5-FU reached 96.3% under NIR irradiation, compared to 88.1% without irradiation within approximately 12 h. Biological evaluation revealed that the drug-free hydrogel exhibited negligible cytotoxicity toward normal LO2 cells, confirming its high biocompatibility. In contrast, the 5-FU-loaded system showed pronounced anticancer activity against SMMC-7721 hepatoma cells, with cell viability decreasing to 28.3% at 512 µg mL^−1^, and further to 21.2% under NIR irradiation, highlighting the synergistic effect of chemotherapy and photothermal therapy.^51^

### 2.2 Tissue engineering

**Fig. 5:**
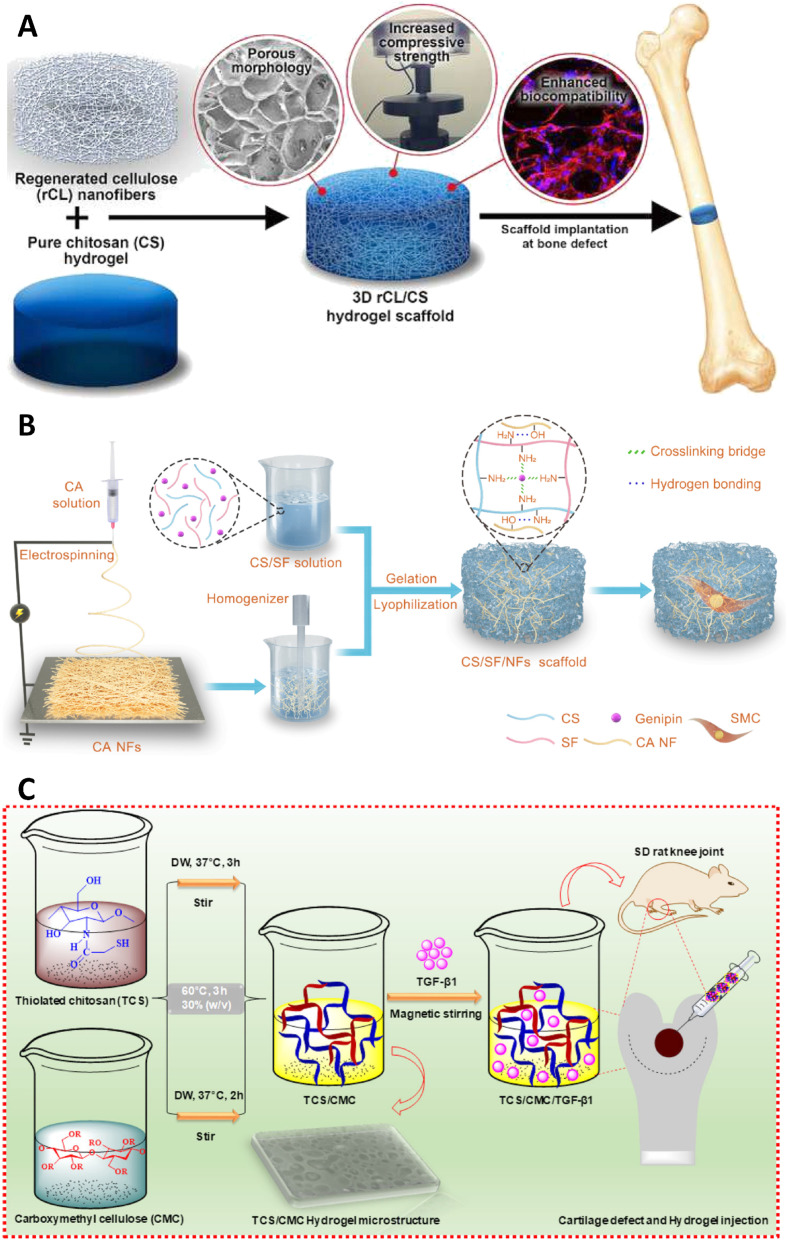
(A) Illustration showing the fabrication of the 3D rCL/CS hydrogel scaffold reinforced with regenerated CNFs and its resulting porous structure and mechanical enhancement. The figure also highlights improved biocompatibility and osteogenic responses, supporting its potential use in bone tissue engineering. Reprinted with permission from ref. 55. Copyright 2020, Elsevier Ltd. (B) Construction of cellulose acetate/SF nanofiber (CA/SF/NFs) composite scaffolds and their subsequent cellularization. Reprinted with permission from ref. 56. Copyright 2022, Elsevier Ltd. (C) The preparation of TGF-β1-loaded TCS/CMC hydrogel systems is depicted. Acellular hydrogels were injected into rat knee joint defects to promote *in vivo* cartilage regeneration. Reprinted with permission from ref. 57. Copyright 2021, Taylor & Francis.

Cellulose–CS (CS–CL) composites have emerged as promising biomaterials for tissue engineering, as well, due to their complementary structural and biological properties. Cellulose provides mechanical strength, porosity, and a stable fibrous framework mimicking the native ECM, while CS contributes biocompatibility, enzymatic degradability, and intrinsic antimicrobial activity. The combination of these polymers results in hydrogels or porous scaffolds with enhanced mechanical stability, controlled degradation, and improved cellular interactions, which are essential for cartilage, bone, and smooth muscle regeneration.^52,53^ Moreover, their tunable physicochemical properties and ability to be combined with proteins or growth factors enable localized delivery of bioactive molecules and stem cells at the defect site, facilitating tissue repair and regeneration.^54^ In fact, CS-CL composites represent a sustainable, bio-based platform for constructing 3D scaffold that effectively mimic ECM features and support functional tissue reconstruction. Recent advancements have emphasized that the reinforcement of natural polymer hydrogels with CNFs to address their inherent mechanical limitations in tissue engineering.^55^ For example, regenerated cellulose (rCL) nanofibers, obtained *via* deacetylation of electrospun cellulose acetate, were incorporated into a chitosan (CS) matrix to fabricate composite hydrogel scaffolds through freeze-induced phase separation followed by freeze-drying (lyophilization) process. In this method, regenerated CNFs were dispersed in a chitosan solution and the resulting mixture was cast into molds, frozen at −80 °C to induce phase separation, and subsequently freeze-dried to obtain a porous scaffold structure. The scaffolds were further neutralized using NaOH solution and washed to remove residual acetic acid prior to final lyophilization (Fig. 5A). The resulting rCL/CS system exhibited a uniform, interconnected porous architecture, in which the nanofiber reinforcement effectively reduced pore size and enhanced overall structural integrity. The incorporation of rCL nanofibers significantly enhanced the compressive strength and cyclic durability of the hydrogel, enabling it to tolerate repeated mechanical loading. This improved resilience is a critical requirement for applications in load-bearing and mechanically demanding tissue engineering scaffolds. Moreover, the composite offered tunable swelling, water absorption, and degradation behavior, enabling modulation of physical stability according to biological requirements. From a biological perspective, the rCL/CS scaffolds promoted pre-osteoblast (MC3T3-E1) cell adhesion, proliferation, and differentiation. Elevated alkaline phosphatase (ALP) activity and increased calcium deposition confirmed their osteoinductive potential. Such improvements arise from the synergistic interaction between the biocompatibility of CS and the reinforcing effect of CNFs, which collectively created a mechanically robust and biologically active environment for bone regeneration. Therefore, the fabricated cellulose–CS composite system represents an effective strategy for reinforcing natural hydrogels through nanofiber incorporation, providing a balanced combination of mechanical strength, biodegradability, and bioactivity. This approach offered valuable insights for designing next-generation biopolymer-based scaffolds in bone tissue engineering and broader regenerative medicine applications.^55^

A biomimetic cryogel scaffold was fabricated *via* multi-step process including electrospinning, mechanical fragmentation of cellulose acetate (CA) nanofibers, solution blending with CS/silk fibroin (SF), and genipin-mediated chemical crosslinking, followed by thermal gelation, freezing, and lyophilization. The resulting scaffolds were further neutralized using NaHCO_3_ solution to remove residual acetic acid and purified by repeated washing and re-lyophilizaon (Fig. 5B). Cryogels inherently possess interconnected macroporous networks suitable for three-dimensional (3D) tissue regeneration; however, their limited mechanical strength often restricts practical use. Incorporating CA nanofibers into CS/SF cryogels provided additional reinforcement and mimicked the natural ECM, thereby enhancing both mechanical integrity and cellular interactions. The addition of electrospun CA nanofibers (average diameter 815.6 ± 192.2 nm) into the CS/SF scaffolds significantly improved their structural and functional properties. The presence of nanofibers increased pore interconnectivity, increasing the average pore size from 216 ± 65.3 µm in pure CS/SF to ∼260 µm in fiber-reinforced scaffolds, while reducing the swelling ratio from 1745.9% to 1048.2% with higher fiber content, reflecting improved structural stability. The incorporation of CA nanofibers significantly enhanced the mechanical properties of CS/SF scaffolds, leading to increased compressive stress and modulus in a concentration-dependent manner while maintaining excellent elasticity and recovery after 70% strain. Notably, the CS/SF/NFs2% scaffold exhibited mechanical properties closer to native smooth muscle tissue and demonstrated good fatigue resistance, retaining over 70% of its maximum stress after 50 compression cycles. Biocompatibility studies demonstrated high cell viability, generally exceeding ∼80%, confirming the non-toxic nature of the scaffold components. Cell proliferation assays showed significantly improved cellular proliferation compared to control samples, attributed to the synergistic effects of chitosan and silk fibroin. Furthermore, the scaffold supported smooth muscle cell alignment and spreading, which are critical for functional tissue regeneration. All of these results confirmed that the developed scaffold had strong potential for applications in smooth muscle tissue engineering.^56^

An injectable hydrogel system composed of thiolated chitosan (TCS) and carboxymethyl cellulose (CMC) was developed *via* a thermally induced self-crosslinking process for transforming growth factor beta-1 (TGF-β1) in cartilage tissue engineering. Hydrogel formation occurred upon incubation at elevated temperature, driven by thiol-mediated interactions and physical network formation, without the use of external chemical crosslinkers (Fig. 5C). The design of such hybrid hydrogel addressed the rapid degradation and short half-life of bioactive molecules under physiological conditions as a major limitation in growth factor therapy. Cross-linking of TCS and CMC under visible light generated a flexible and stable 3D network suitable for minimally invasive injection and localized delivery. The TCS/CMC hydrogel exhibited a porous structure and strong viscoelastic behavior, providing structural integrity while maintaining high water retention and gradual biodegradation. The swelling ratio reached up to 177.4 ± 8.8%, while equilibrium water loss ranged between 82.4 ± 4.1% and 93.2 ± 4.6%. The hydrogels maintained over 90% of their original weight after 21 days, indicating slow and controlled degradation. TGF-β1 released pattern showed an initial burst release (48.6 ± 2.4% on day 1), followed by sustained release up to ∼81.4 ± 4.0% over 21 days, suggesting efficient retention and controlled delivery. *In vitro* and *in vivo* evaluations demonstrated that such systems effectively enhance bone marrow stem cell (BMSC) proliferation and chondrogenic differentiation, leading to significant restoration of cartilage tissue in defect models.^57^

Mimicking the hierarchical organization of native cartilage extracellular matrix (ECM), a composite hydrogel scaffold was developed by integrating a chitosan (CS)–collagen hydrogel with 3D-printed poly(lactic acid) (PLA) lattices and wet-electrospun CNFs. In this multi-step fabrication strategy, PLA frameworks were first produced *via* 3D printing, followed by the deposition of CNFs using a wet electrospinning technique. These reinforcing components were subsequently incorporated into the CS–collagen hydrogel matrix and chemically crosslinked using genipin (0.1–0.5%), resulting in a structurally integrated composite scaffold obtained *via* freeze-drying. In here, the integration of PLA microstructures and CNFs within the hydrogel matrix provided both mechanical reinforcement and bioactive support for cellular activities. The scaffolds cross-linked with various concentrations of genipin (0.1–0.5%) exhibited an interconnected microporous structure with high swelling capacity (∼400%) and water content (77–83%), closely resembling natural cartilage. Among them, the formulation containing 0.3% genipin achieved the most balanced performance, demonstrating optimal compressive strength (∼32 kPa) and viscoelasticity suitable for cartilage regeneration. *In vitro* evaluations confirmed excellent cytocompatibility, as rabbit mesenchymal stem cells readily attached, proliferated, and migrated throughout the scaffold. Moreover, chondrogenic differentiation was evidenced by the upregulated expression of type I and II collagen and the formation of chondrocyte-like cells. According to these results, the synergistic integration of CS, collagen, and cellulose with a 3D-printed PLA framework yielded a composite scaffold with superior mechanical integrity and biological functionality, emphasizing its strong potential for meniscus cartilage regeneration.^58^

3D-printed hydrogels have also been explored for fabricating scaffolds suitable for mechanically demanding soft tissues such as intervertebral discs, cartilage, and meniscus. In this context, a composite hydrogel based on natural chitosan (CHI) and CNFs was developed using a direct ink writing (DIW) extrusion-based 3D bioprinting process, where shear-thinning CHI/CNF bioinks were extruded through a pressure-controlled nozzle and deposited layer-by-layer into a sodium hydroxide (NaOH) coagulation bath. In this system, *in situ* ionic neutralization of chitosan induced rapid hydrogel solidification, enabling the formation of free-standing 3D architectures without the need for chemical crosslinkers. The optimized CHI/CNF formulations enabled precise filament deposition and stable construct formation, achieving high printing resolution and structural fidelity. Incorporation of CNFs notably enhanced the mechanical robustness of the hydrogel while preserving the hydrophilic and cytocompatible nature of CS. The printed hydrogels exhibited an anisotropic microstructure and supported extensive 3D-cell colonization, indicating favorable conditions for tissue integration. Rheological analysis further revealed shear-thinning behavior suitable for smooth extrusion and consistent scaffold fabrication. By adjusting the polymer ratio and printing parameters, the system achieved a well-balanced combination of printability, mechanical performance, and biological functionality. These findings highlighted the potential of purely natural CHI/CNF composites as mechanically resilient and biologically active 3D-printed hydrogels. Their ability to mimic the hierarchical and anisotropic features of native soft tissues suggested promising applications in tissue engineering and the future design of patient-specific, bioinspired implants.^59^

**Fig. 6:**
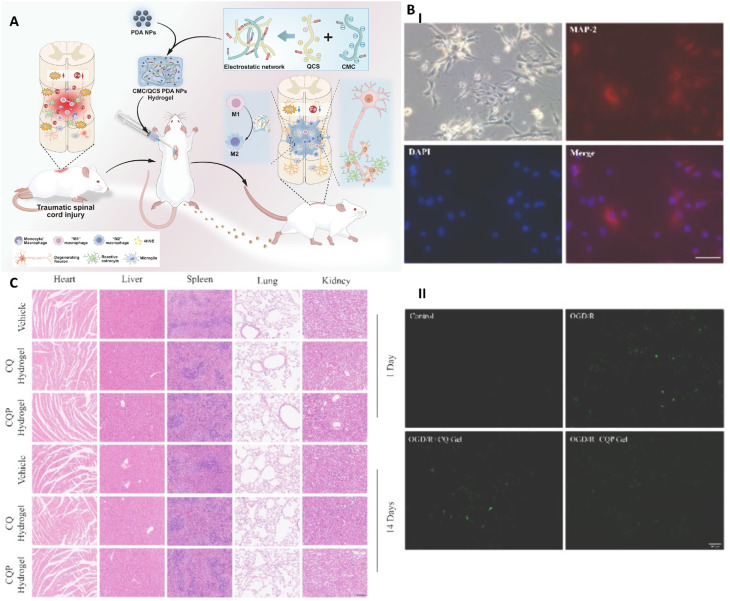
(A) Mechanism by which the injectable ROS-scavenging hydrogel with enhanced anti-inflammatory activity promotes spinal cord repair through ferroptosis inhibition. (B) Immunofluorescence analysis of primary spinal cord neurons. Neuronal cell bodies and processes were labeled with MAP2 antibody (red), nuclei were stained with DAPI (blue), and the merged image shows the overlay of MAP2 and DAPI signals. Scale bar: 50 µm. II: Cellular ROS levels following OGD/R treatment were assessed using DCFH-DA staining and quantified by flow cytometry. Data are presented as mean ± SD, *n* = 3 per group. (C) Representative H&E-stained sections of major organs from rats at 1 and 14 days post hydrogel injection. Scale bar: 200 µm. Reprinted with permission from ref. 60. Copyright 2024, Elsevier B.V.

Spinal cord injury (SCI) remains a disabling neurological disorder with limited therapeutic options for functional recovery. Recent studies have highlighted ferroptosis, a regulated form of iron-dependent cell death, as a key contributor to neuronal damage following SCI. To address this challenge, a functional hydrogel system (Fig. 6) was developed using CMC and quaternized CS (QCS) incorporated with polydopamine nanoparticles (PDA NPs) to create a multifunctional composite (CQP hydrogel). In details, hydrogel was fabricated *via* a polyelectrolyte complexation-based hydrogel formation between CMC and QCS. Separately, PDA NPs were synthesized through alkaline self-polymerization of dopamine followed by EDC/NHS-mediated PEG functionalization. The PDA NPs were subsequently incorporated into the CMC/QCS hydrogel precursor prior to gelation to obtain a nanoparticle-loaded hydrogel system. The hydrogel exhibited a stable and highly porous three-dimensional structure, which facilitated efficient drug/nanoparticle distribution and supported cellular infiltration. Utilizing PDA NPs improved the mechanical strength and antioxidative properties of the system, contributing to enhanced structural stability and biological functionality. The composite hydrogel demonstrated significant neuroprotective effects by effectively inhibiting ferroptosis, as evidenced by reduced intracellular reactive oxygen species (ROSs) levels and decreased lipid peroxidation (about 40–60% reduction), and other markers associated with ferroptosis. In addition, the system modulated macrophage polarization, promoting a shift from the pro-inflammatory M1 phenotype to the anti-inflammatory M2 phenotype. *In vitro* and *in vivo* evaluations revealed that the hydrogel significantly enhanced neuronal survival and axonal regeneration. Functional recovery assessments indicated marked improvement, with locomotor scores increasing by approximately 30–50% compared to control groups over the treatment period. Furthermore, the hydrogel exhibited good biocompatibility, with cell viability above 85%, confirming its safety for biomedical applications.^60^ This study introduced a bioinspired, iron-chelating hydrogel platform that not only inhibits ferroptosis but also reprograms immune responses to enhance spinal cord repair. The dual-action mechanism-ferroptosis suppression and macrophage modulation-marked a significant advancement in the development of biomaterial-based therapies for SCI recovery.

### 2.3 Wound healing

Wound healing is a complex and dynamic biological process involving hemostasis, inflammation, proliferation, and remodeling phases. Inefficient healing can lead to chronic wounds, which remain a major clinical challenge and significantly affect quality of life of patients. Thus, developing advanced materials and therapies to promote faster and more effective wound repair is of great importance.^61^ Hydrogels have emerged as one of the most promising biomaterials for wound management due to their high water content, biocompatibility, and structural similarity to the natural ECM. They can provide a moist environment, promote gas exchange, and act as a protective barrier against microbial invasion, all of which facilitate faster tissue regeneration. Among natural polymer-based hydrogels, cellulose–CS biocomposites have gained particular attention. The combination of excellent mechanical stability of cellulose with intrinsic antibacterial and hemostatic properties of CS results in dressings that not only protect the wound but also actively support cell proliferation, angiogenesis, and collagen deposition. Recent studies confirmed the effectiveness of cellulose–CS hydrogels in accelerating wound closure, reducing inflammation, and enhancing granulation tissue formation, making them highly suitable for both acute and chronic wound applications.^2,22,62^ For instance, a nanogel-based system was introduced in which a polymeric carrier was combined with bioactive oils, such as *Nigella sativa* oil, that act as natural permeation enhancers while contributing additional antibacterial, antioxidant, and anti-inflammatory effects. Such integration not only facilitated transdermal drug penetration across the stratum corneum but also accelerated tissue regeneration through modulation of fibroblast activity and upregulation of key wound-healing mediators including Fibroblast growth factor 2) FGF2, (vascular endothelial growth factor)VEGF, (and Transforming growth factor beta (TGF-β1). This study showed that (atorvastatin-oil nanogel) ATONG-type nanogels maintain excellent cytocompatibility with fibroblasts and minimal hemolytic activity, supporting their safety for topical applications. Moreover, their nanoscale structure allowed controlled and sustained drug release while simultaneously protecting the wound site from bacterial infection, particularly from *Staphylococcus* species. Therefore, these characteristics highlighted the promise of *Nigella sativa*-enriched atorvastatin nanogels as dual-function wound healing systems that combine efficient transdermal delivery, anti-infective protection, and cellular modulation-features that highlight their potential for future clinical translation.^36^

TCS and oxidized CMC (OCMC), integrated with Cu-doped borate bioactive glass (BG) were used to produce an *in situ* forming hydrogel for wound healing application. Thiolation of CS enhanced its solubility and mucoadhesive properties, while dynamic Schiff base linkages enabled the formation of an injectable and self-crosslinking network. The incorporation of borate BG provided dual functionality-antibacterial effects and stimulation of angiogenesis-through the controlled release of Cu^2+^ and B^3+^ ions. *In vitro* evaluations revealed that the hydrogel exhibited strong antibacterial activity against both *Staphylococcus aureus* (*S. aureus*) and *Escherichia coli* (*E. coli*), attributed to the synergistic effects of cationic nature of CS and the released copper ions from borate BG, which disrupt bacterial membranes and inhibit biofilm formation. Cytotoxicity assays using fibroblast and keratinocyte cell lines confirmed good biocompatibility, with enhanced cell attachment and proliferation on the hydrogel surface, confirming the cytocompatibility and cell-supportive nature of the fabricated hydrogel. *In vivo* studies using a full-thickness wound model in mice further validated the therapeutic potential of the developed hydrogel. The Cu-doped borate glass-loaded hydrogel (OCMC/TCh/BG) maintained structural integrity at the wound site, promoted angiogenesis, and accelerated epithelial regeneration compared to the control and BG-free hydrogel groups. Histological analysis after 14 days showed increased collagen deposition, reduced inflammation, and formation of mature granulation tissue, confirming the synergistic effects of the polysaccharide matrix and bioactive glass on tissue remodeling. Therefore, borate-containing TCh/OCMC hydrogels were considered as promising bioactive dressings, offering an injectable, cost-effective, and growth factor-free strategy for promoting tissue regeneration and angiogenesis in skin wound healing.^63^

Chronic diabetic wounds represent a significant therapeutic challenge due to prolonged inflammation and suppressed tissue regeneration under hyperglycemic conditions. To overcome these obstacles, recent studies have investigated advanced hydrogel systems composed of natural polymers such as CS and gelatin, reinforced with CNFs to improve mechanical strength and moisture retention.^64^ In an innovative approach, a nanoemulsion of oregano essential oil, a natural antibacterial and anti-inflammatory compound, was incorporated into the hydrogel network to form a composite nanoemulgel system. The CS-coated oregano essential oil nanoemulsion, embedded within a gelatin–polyvinylpyrrolidone hydrogel reinforced with CNFs, provided enhanced stability, bioavailability, and a sustained drug release profile within the polymeric matrix. This hybrid hydrogel exhibited excellent swelling ability, mechanical strength, and fibroblast compatibility, supporting efficient cell adhesion and proliferation. *In vivo* assessments on diabetic rat models demonstrated accelerated wound healing, reduced inflammation, enhanced collagen remodeling, and nearly complete epithelial regeneration. Additionally, the integration of photobiomodulation using low-level laser therapy acted synergistically with the bioactive hydrogel, further stimulating fibroblast proliferation and vascularization, resulting in almost complete wound closure within 48 h. This combined approach highlighted the potential of multifunctional, naturally derived hydrogels coupled with light-assisted therapies as an advanced platform for supporting normal healing dynamics in chronic diabetic wounds. Moreover, these systems hold promise for future integration into microneedle patches and 3D bioprinting platforms, expanding their applicability in next-generation wound care.^64^

Uncontrolled internal bleeding remains a major cause of trauma-related mortality, highlighting the importance of designing biomaterials capable of achieving rapid hemostasis, preventing infection, and maintaining structural stability under physiological stress. A cryogel composed of QCS and oxidized BC (OBC) was produced to overcome these challenges, which combined mechanical resilience with bioactivity through dual crosslinking strategies. The QCS/OBC cryogel, prepared *via* genipin-mediated covalent crosslinking and electrostatic interactions, formed a flexible, macroporous 3D network with rapid shape-memory recovery within 2 s and a nearly 100% recovery ratio. Its interconnected porous structure allowed efficient blood absorption and platelet aggregation, promoting coagulation, while CS promoted hemostasis and the quaternary ammonium groups enabled stable antibacterial activity. The cryogel showed good flexibility and rapid re-expansion, allowing it to cover deep or hard-to-compress wounds without external pressure. *In vivo* experiments in rat liver models demonstrated superior hemostatic performance, with low blood loss and rapid hemostasis times of 20–50 s. Cytocompatibility and histological analyses further confirmed the biocompatibility of fabricated formulation with enhanced microvessel formation at the wound site and lack of adverse effects. Therefore, the QC/OBC cryogel could combine rapid shape recovery, strong pro-coagulant activity, antibacterial properties, and tissue compatibility, making it a highly promising material for emergency treatment of noncompressible hemorrhage.^65^

**Fig. 7:**
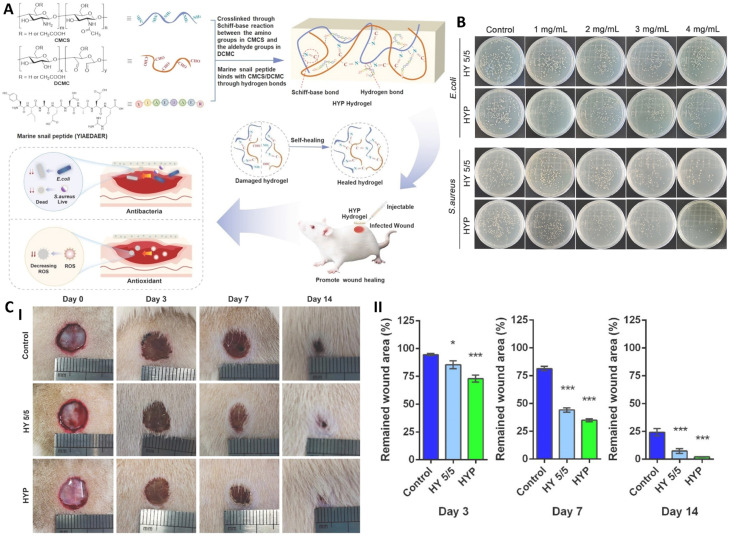
(A) Synthesis of the injectable, self-healing HYP hydrogel designed for the treatment of infected wounds. (B) Representative images showing surviving bacterial colonies on agar plates. (C) (I) Photographs of rat wounds captured on days 0, 3, 7, and 14, illustrating progressive closure over time. (II) Quantitative analysis of remaining wound areas on days 3, 7, and 14 in different treatment groups. Data are presented as mean ± SD. Statistical significance: *p < 0.05, ***p < 0.001 *versus* Control. Wound area at day 0 was set as 100% for normalization. Reprinted with permission from ref. 66. Copyright 2024, Elsevier B.V.

Bacterial infection remains a major inhibition against effective wound healing, as it sustains chronic inflammation and promotes progressive tissue necrosis. This led to the fabrication of a peptide-functionalized polysaccharide hydrogel (Fig. 7) that combined antimicrobial, antioxidant, and regenerative properties. This hydrogel was composed of CMCS and dialdehyde CMC (DA-CMC), crosslinked *via* dynamic Schiff-base reactions and functionalized with a marine snail-derived peptide (YIAEDAER). This formulation resulted in an injectable, self-healing hydrogel (HYP) with balanced mechanical and biological characteristics appropriated for infected full-thickness wounds. The injectable and self-healing HYP hydrogel, reinforced with reversible imine bonds, allowed rapid recovery and easy application while maintaining structural integrity. Its porous 3D network supported hydration, nutrient diffusion, and a moist microenvironment favorable for tissue repair. The marine snail peptide YIAEDAER significantly enhanced antioxidant activity and inhibited bacterial growth (*S. aureus* and *E. coli*) *in vitro*. In bacterial-infected full-thickness skin defect rat models, the hydrogel accelerated wound closure, reducing the wound area to 2% by day 14, promoted collagen deposition, organized fibroblasts, hair follicle formation, and increased angiogenesis markers (CD31 and alpha-smooth muscle actin (α-SMA)), while modulating inflammation (upregulated interlukine-10 (IL-10), downregulated IL-6). Moreover, the hydrogel demonstrated low hemolytic activity (0.74%) and good cytocompatibility, highlighting its potential as a multifunctional dressing that combines self-healing, antibacterial, antioxidant, and tissue-regenerative properties.^66^

**Fig. 8:**
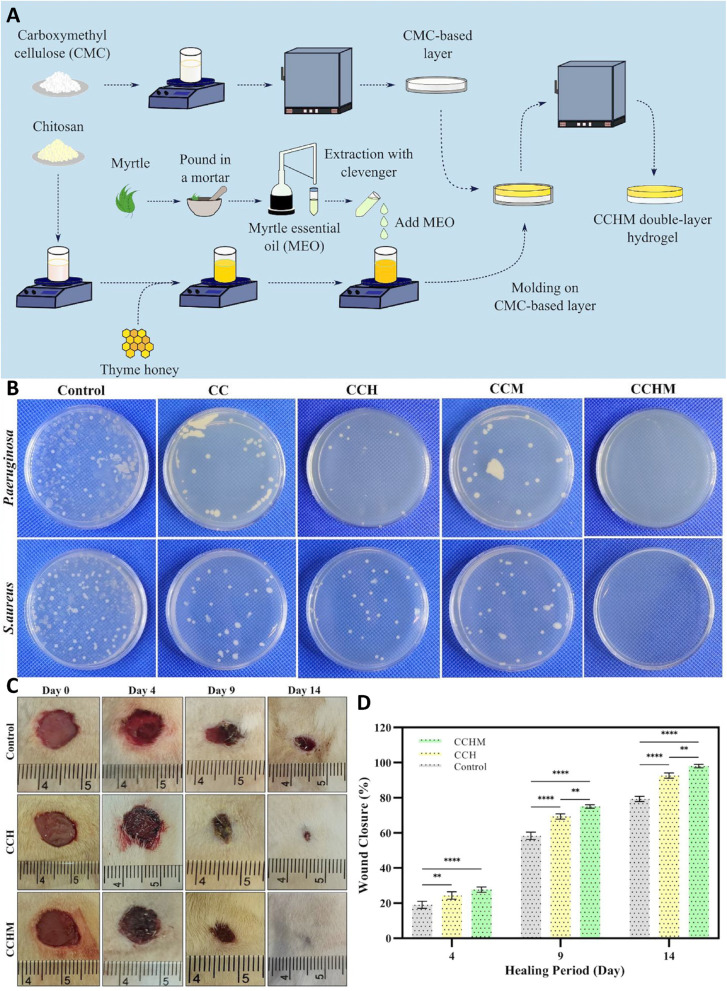
(A) Design and preparation steps of the double-layer hydrogel system. (B) Evaluation of antibacterial activity of the double-layer hydrogels *via* the minimum inhibitory concentration (MIC) assay. (C) *In vivo* wound healing assessment using CCH and CCHM double-layer hydrogels. (D) Representative images of wounds treated with Control, CCH, and CCHM samples on days 0, 4, 9, and 14. * indicates a statistically significant difference, *P* < 0.05. Data are presented as mean ± SD (*n* = 3). Reprinted with permission from ref. 67. Copyright 2023, Elsevier B.V.

Bacterial contamination and oxidative stress are among the major barriers to efficient wound repair, as they hinder tissue regeneration and prolong inflammation. In one study, CMC/CS-based double-layer hydrogel incorporating natural bioactive agents myrtle essential oil (MEO) and thyme honey was produced to overcome these limitations (Fig. 8). In here, the outer CMC layer formed a smooth, semi-permeable barrier that limited microbial infiltration, while the CS layer enriched with honey and essential oil was responsible for antioxidant and antimicrobial activity. The synergistic combination of these natural components enhanced free-radical scavenging, reduced bacterial colonization, and supported cell adhesion and proliferation, thereby promoting a favorable environment for wound regeneration. *In vitro* studies showed fibroblast viability of over 85%, significant antibacterial activity with up to 96% reduction in pathogenic bacteria, and enhanced blood clotting compared to control groups. *In vivo* results confirmed the beneficial effect of the hydrogel on accelerating the wound closure, achieving nearly 98% closure within 14 days, with enhanced collagen deposition and reduced inflammation. These results highlighted the potential of this hydrogel as a multifunctional wound dressing with antimicrobial, antioxidant, and hemostatic properties. Due to their natural composition, biocompatibility, and hemostatic potential, such bilayer hydrogels exemplify sustainable and multifunctional wound dressings that bridge traditional natural remedies with contemporary biopolymer engineering.^67^

Smart hydrogels, such as thermosensitive ones, have recently attracted increasing attention for wound management due to their ability to undergo sol-to-gel transition at physiological temperatures, enabling easy application and *in situ* conformability to irregular wound surfaces.^68^ In this context, a phase-transition hydrogel system composed of decanoic acid-modified CS (CSDA) and methyl cellulose (MC) was produced *via* temperature-induced hydrophobic interactions. This CSDA-MC hydrogel (CSDA-MC-HG) exhibited favorable viscoelastic and shear-thinning behavior, allowing smooth injection, uniform spreading, and stable adhesion to wound sites. Its interconnected porous network supported moisture retention, oxygen permeability, and nutrient diffusion, conditions that are essential for optimal tissue regeneration. Physicochemical characterization confirmed that hydrogen bonding and electrostatic interactions controlled the gelation process, ensuring structural stability without compromising biocompatibility. *In vitro* assays demonstrated excellent cytocompatibility, hemocompatibility, and fibroblast proliferation, while *in vivo* studies revealed enhanced wound closure, angiogenesis, collagen deposition, and re-epithelialization.^69^ Therefore, this thermosensitive CSDA–MC hydrogels demonstrated a promising class of injectable and adaptive biomaterials that integrated ease of application with robust biological performance for efficient skin tissue repair.

We have summarized some important examples of cellulose–CS based hydrogels in biomedical applications in Table 1.

**Table 1:** Some important examples of cellulose–CS based hydrogels and their biomedical applications

Material composition	Preparation method/crosslinking	Structural/functional features	Therapeutic agents	Findings	Ref.
Aldehyde-functionalized cellulose + CS	Schiff base (aldehyde-amine condensation)	Biodegradable, pH-sensitive swelling, controlled drug release	Streptomycin	DAC ratio controls release rate; hydrogels non-toxic to fibroblasts; molecular docking indicates strong interactions between DAC, CS, and streptomycin	70
Sodium alginate + CS + cellulose nanofibrils (CNF)	Physical crosslinking *via* CNF reinforcement	Enhanced mechanical strength, reduced swelling, sustained release	Model drug (unspecified)	1% CNF increased storage modulus 23.6% and loss modulus 54.4%; release rate in gastric juice reduced by 50.8%, showing effective sustained release	71
BC + CS + silver sulfadiazine	Chemical crosslinking with glutaraldehyde	pH-sensitive, antibacterial, mechanically stable	Silver sulfadiazine	Significant improvement in antibacterial activity, especially against *S. aureus*; maintained microporous structure and swelling behavior	72
CS + CMC	Polyelectrolyte complexation	Ionic interaction, sustained release, matrix stability	Clarithromycin	Ionic interactions enhanced drug entrapment; release controlled by Fickian diffusion; varying CS/CMC ratios modulate release kinetics	73
Mesoporous silica (MSN) + sodium hyaluronate + CS + oxidized CMC	Schiff base + disulfide crosslinking	Dual-responsive (pH & GSH), dual-drug delivery, intelligent release	Cytarabine + methotrexate	Dual-drug hydrogel showed higher cytotoxicity than single-drug; responsive release triggered by acidic pH and glutathione presence	50
GO + hydroxypropyl cellulose + CS	Glutaraldehyde crosslinking	Improved drug loading, reduced burst release, pH-sensitive	5-FU	GO improved encapsulation efficiency and release controllability; drug release influenced by pH and GO content	74
CS + cellulose + polyacrylamide (double-network, CCP hydrogel)	Chemical + physical double-network	High compressive strength, high swelling ratio, water retention, selective adsorption	Tetracyclines (tetracycline, minocycline, demeclocycline)	CCP hydrogel 2.8× stronger than single network; good adsorption capacity (84–93% retention after cycles); high accuracy in detecting tetracyclines in food	75
BC + CS (zwitterionic hydrogel)	Schiff base (aldehyde-amine)	pH-responsive, good mechanical stability, high drug loading	Naproxen	Swelling sensitive to pH (minimum at 3.5–5.0); sustained release >24 h; release followed zero-order kinetics; potential for clinical therapy	76
Cellulose nanocrystals (CNC) + CS	Glutaraldehyde crosslinking	pH-sensitive, swelling responsive, osteoblast-compatible	5-Aminosalicylic acid	Release optimized at pH 7.4 (intestinal); inhibited under acidic pH (gastric); Korsemeyer–Peppas kinetics; biocompatible with osteoblasts	77
Alginate, methyl cellulose (MC), trimethyl CS (TMC), silicate glasses	3D printing	TMC improved scaffold stability, bone tissue engineering	—	TMC enhances scaffold stability, supports mineralization	78
BC, CS, alginate, gelatin	Hydrogel mixing + CaCl_2_ crosslinking	Good compressive strength	—	Multi-polymer composite, supports 3D cell growth	79
CS, pluronic, gold-decorated oxidized CNFs (CS/pluronic/Au–OCNF)	Injectable hydrogel/thermosensitive physical gelation	Shear modulus 1–12 kPa	—	Electroconductive, injectable, thermoresponsive	80
CS hydrogel with kenaf nanocrystalline cellulose (NCC) and platelet lysate (PL)	NCC incorporated into CS hydrogel	High water retention, prolonged PL release, fibroblast proliferation	Platelet lysate (PL)	Enhanced growth factor stability and wound closure *vs.* CS alone	81
				Wound healing (*in vitro* scratch test)	
BC with CS polymer or nanoparticles (nChiD)	Composite hydrogels with gamma-irradiated CS	High porosity, absorption, *anti*-biofilm, biocompatibility	—	Chronic wound dressings (*anti*-biofilm, cell migration)	82
				BC–nChiD: best healing rate, strong *anti*-biofilm activity	
Oxidized microcrystalline cellulose + carboxymethyl CS	Schiff-base (aldehyde–amine) crosslinking	Self-healing, pH-sensitive, hemostatic, drug release	Rutin (a drug model)	Wound dressing & drug delivery; good coagulation; controlled drug release; bioactive wound care	83
CMCS-Hep + CMC-A	Schiff-base reaction	Injectable, biodegradable, self-healing, pH-responsive; dual-drug loading	Superoxide dismutase (SOD*)* and recombinant human epidermal growth factor (rhEGF*)*	Improved wound closure and accelerated tissue regeneration	84
				Diabetic wound healing; dual-drug delivery	
CMCS + CMC microspheres (tetracycline)	Schiff-base hydrogel; emulsion crosslinking for microspheres	Elastic, self-healing, sustained antibiotic release, biodegradable	Tetracycline hydrochloride (TH)	Sustained tetracycline release; enhanced antibacterial performance	85
				Wound dressing with sustained drug delivery	
Aldehyde-functionalized CNFs + CS	Schiff-base crosslinking; cryogelation	Self-healing (∼100%), shear-thinning, shape-memory cryogel, high water uptake	—	Injectable shape-memory scaffold; large water absorption	86
				Biomedical scaffolds; tissue engineering	
CMCS reinforced with s-NC	Physical blending & crosslinking	Injectable, adhesive, conductive, porous	Growth factors	Improved adhesion and antibacterial performance	87
				Rapid skin regeneration dressing	
HPCS-g-SFP + OMCC with TMP	Schiff-base linkage	Injectable, tunable pore size, antioxidant, anti-scarring	Tetramethylpyrazine (TMP)	Reduced scarring; enhanced wound healing	88
				Scarless wound healing applications	
CS, CMC-g-PF127, nano-curcumin	Solvent casting & graft copolymerization	Thermoresponsive, viscoelastic, controlled release, good swelling	Curcumin	Prolonged curcumin retention; accelerated diabetic wound repair	89
				Diabetic wound therapy with nano-curcumin	
BC impregnated with fungal CMCS	Solution immersion impregnation	Improved hydrophilicity, enhanced cell attachment, antibacterial groups present	—	Enhanced antibacterial activity and cell colonization	90
				Antibacterial wound dressing based on BC	
Oxidized regenerated cellulose (ORC) + CS + EDA-CD + ZnO NPs + IBU	RC oxidation; assembly with CS and EDA-CD; incorporation of ZnO and IBU complex	pH-responsive degradability; sustained ibuprofen release; antimicrobial ZnO inclusion	Ibuprofen (IBU)	Sustained analgesic release and antimicrobial protection; pH-responsive analgesic and antimicrobial wound dressing	91
CS with reduced GO (rGO) + PCL/CA electrospun membrane	Hydrogel with rGO; electrospinning of PCL/CA to form asymmetric dressing	NIR-responsive photothermal effect; moist hydrogel layer with protective nanofiber barrier	Dopamine hydrochloride	Mild photothermal antibacterial therapy; supports skin regeneration	92
				Asymmetric photothermal-responsive wound dressing	
Dialdehyde CMC (DCMC) + dopamine-modified CMCS (CS-DA)	Schiff-base dynamic crosslinking	Robust tissue adhesion, self-healing, on-demand detachment, injectable	Dopamin	Strong wet adhesion and convenient removal; shape-adapting filling of scald wounds	93
				Deep burn wound dressing with controlled detachment	
Allyl cellulose (AC) + CMCS dual network	Hygroscopic physical adhesion followed by covalent amine crosslinks	Ultrafast adhesion on wet tissue, antibacterial, hemostatic, tunable mechanics	—	Ultrafast wound sealing and improved hemostasis *vs.* commercial film	94
				Emergency wound management; first-aid adhesive hydrogel tape	
CMC + CS + PVA crosslinked by citric acid and functionalized with l-arginine	Citric acid esterification crosslinking; l-Arg biofunctionalization	3D porous, hydrophilic, bioadhesive scaffold with enhanced cell-adhesion sites	—	Enhanced cell colonization, bioadhesion, accelerated skin repair *in vivo*	95
				Bilayer skin substitute for cell therapy and wound healing	

## 3 Challenges and future perspectives

Despite their promise in stimuli-responsive therapeutics, CS–cellulose hydrogels face significant challenges in achieving widespread clinical use. Key challenges include mechanical weaknesses, scalability issues, and regulatory barriers that slow translation from lab to patient care. These hydrogels often have low mechanical stability, leading to structural failure under stress, and poor durability and unpredictable biodegradation can compromise long-term performance. Additionally, variations in polymer composition and crosslinking density result in inconsistent swelling behavior and drug release profiles. Recent studies have highlighted that the physicochemical properties and stability of cellulose–CS hydrogels strongly depend on network architecture and dynamic crosslinking mechanisms.^96^ Moreover, the thermal stability of CS–cellulose hydrogels under hyperthermia conditions remains an underexplored but critical challenge, particularly for stimuli-responsive and photothermal therapeutic applications. Elevated temperatures can disrupt hydrogen bonding and weaken intermolecular interactions within the hydrogel network, leading to structural instability, accelerated degradation, and altered swelling behavior. Studies have shown that temperature-responsive chitosan-based hydrogels undergo significant physicochemical changes at elevated temperatures, including phase transitions and modifications in heat transfer and stability properties.^97^ These temperature-induced changes may result in uncontrolled drug release and reduced mechanical integrity, especially in systems exposed to NIR irradiation or other external stimuli. Therefore, improving thermal resistance through optimized crosslinking strategies and incorporation of thermally stable components is essential for enhancing the reliability and clinical applicability of CS–cellulose hydrogels in advanced biomedical systems. Furthermore, fabrication techniques such as freeze-drying and electrospinning frequently produce heterogeneous structures, limiting reproducibility and scalability. Batch-to-batch variability in natural polymer sources further complicates standardization and regulatory approval. Challenges related to biocompatibility, immune response, and long-term *in vivo* stability also remain significant barriers. Additionally, clinical translation interrupted due to the insufficient long-term preclinical data, allergic potential, and challenges in proving multifunctionality under physiological conditions. Wound dressing applications are limited by exudate absorption and gaseous exchange, which may hinder clinical approval processes. Furthermore, the integration of personalized stimuli-responsive features necessitates more comprehensive *in vivo* validation to ensure safety and efficacy. Following implantation, CS–cellulose hydrogels are susceptible to protein fouling and biofilm formation, which can diminish their long-term antibacterial performance despite the intrinsic antimicrobial properties of chitosan. In tissue engineering applications, inadequate vascularization within thick scaffolds restricts the diffusion of oxygen and nutrients, thereby compromising cell viability and tissue regeneration. Additionally, differences between animal models and human physiological responses, such as variations in degradation behavior influenced by immune and metabolic factors, pose significant translational challenges. The precise tuning of responsiveness to environmental stimuli, including pH, temperature, and light, remains difficult to standardize across batches, often resulting in inconsistent drug release profiles under varying physiological conditions. Moreover, externally applied triggers such as NIR irradiation require careful optimization of energy input to prevent unintended tissue damage, while multi-stimuli-responsive systems may experience signal interference or crosstalk, ultimately reducing therapeutic efficacy. From a translational and industrial perspective, the economic feasibility and large-scale manufacturability of CS–cellulose hydrogels remain critical challenges that are often overlooked. Although both chitosan and cellulose are abundant and relatively low-cost biopolymers, the overall production cost of advanced hydrogel systems can increase significantly due to purification processes, chemical modifications, and incorporation of functional nanomaterials. In addition, scaling laboratory-scale synthesis to industrial production introduces challenges related to process reproducibility, quality control, and compliance with Good Manufacturing Practice (GMP) standards.^98,99^ Advanced fabrication approaches, such as multi-stimuli-responsive systems and 3D/4D bioprinting technologies, while scientifically promising, may further limit cost-effectiveness and scalability for commercial applications. Furthermore, issues related to sterilization, storage stability, and shelf life must be carefully addressed to ensure successful market translation. Despite these limitations, the growing demand for sustainable, biocompatible, and multifunctional biomaterials in drug delivery, wound healing, and tissue engineering suggests a strong commercialization potential. Therefore, future research should prioritize the development of cost-effective synthesis routes, scalable manufacturing strategies, and standardized production protocols to facilitate the transition of CS–cellulose hydrogels from laboratory research to clinical and industrial applications. To better summarize the translational potential and limitations of these systems, a SWOT analysis of CS–cellulose hydrogels in biomedical therapeutics is presented in Table 2.

**Table 2:** SWOT analysis of chitosan–cellulose hydrogels for biomedical therapeutics

Catogory	Discription	Ref.
Strengths	Excellent biocompatibility and biodegradability due to the natural polysaccharide origin of chitosan and cellulose	30 and 100
	Tunable physicochemical properties through chemical modification	
	Intrinsic antimicrobial activity of chitosan	
	Strong hydrogen-bonding interactions enabling formation of stable hydrogel networks suitable for drug delivery, wound healing, and tissue engineering	
Weeknesses	Limited mechanical strength and structural stability under physiological conditions	101
	Uncontrolled degradation kinetics	
	Variability in molecular weight and degree of deacetylation of chitosan affecting reproducibility	
	Potential cytotoxicity associated with certain crosslinkers or residual reagents	
Opportunities	Development of multi-stimuli-responsive hydrogels (pH, enzyme, temperature) for targeted drug delivery	30 and 102
	Integration with nanomaterials such as graphene, metallic nanoparticles, and magnetic nanoparticles to enhance mechanical and therapeutic performance	
	Incorporation into smart wound dressings, biosensors, and regenerative scaffolds	
Threats	Regulatory challenges for clinical translation	101 and 102
	Batch-to-batch variability in natural polymer sources affecting scalability	
	Insufficient long-term *in vivo* safety data	
	Competition with synthetic smart polymers that provide more predictable mechanical and degradation properties	

From an environmental sustainability perspective, life cycle assessment (LCA) represents an essential framework for evaluating the environmental impacts of chitosan–cellulose hydrogels throughout their production, use, and end-of-life stages. LCA is widely applied to quantify environmental burdens across the full life cycle of biopolymeric materials, including raw material extraction, processing, application, and disposal, particularly in the context of sustainable biomaterials development.^103^ Although chitosan and cellulose are renewable and biodegradable polymers, their overall environmental footprint depends not only on their natural origin but also on extraction procedures, chemical modifications, crosslinking reactions, and energy-intensive fabrication methods. Recent life cycle studies on cellulose-based materials have demonstrated that processing energy, end-of-life management, and waste treatment significantly influence total environmental impact.^104^ Furthermore, sustainable development of polymeric biomaterials requires optimization of production efficiency, reduction of hazardous reagents, and integration of green chemistry principles to minimize environmental burden during manufacturing. While polysaccharide-based systems generally demonstrate improved biodegradability compared to petroleum-derived polymers, systematic LCA evaluations remain limited for many advanced hydrogel formulations, emphasizing the need for comprehensive environmental impact assessments to support scalable and industrial translation of CS–cellulose hydrogels.^105^ Therefore, integrating LCA methodologies into future research will help optimize eco-friendly synthesis routes, reduce energy consumption, and improve the overall sustainability profile of CS–cellulose hydrogel technologies for clinical and industrial applications.

Researchers are increasingly focusing on developing multi-stimuli-responsive systems that can adapt to complex physiological environments. For instance, hydrogels capable of responding to multiple triggers, such as pH, temperature, and enzymes, could enable highly targeted drug delivery, minimizing side effects, and maximizing therapeutic efficiency. Additionally, the incorporation of nanomaterials and bioactive compounds will likely enhance their functionality, allowing for personalized treatments that can dynamically adjust to individual patient needs. Moreover, advancements in bioprinting technologies are poised to develop the fabrication of these hydrogels, enabling the creation of customized, tissue-specific scaffolds for regenerative medicine. Integrating 4D printing techniques—where hydrogels evolve in shape, stiffness, and porosity over time in response to biological cues—promises patient-specific constructs for cardiac patches or neural interfaces that self-assemble post-implantation, surpassing traditional 3D bioprinting limitations. Furthermore, sustainability and scalability are gaining importance, prompting a shift toward greener synthesis methods and eco-friendly production processes. Future research will probably emphasize the development of cost-effective, environmentally conscious manufacturing techniques that can be translated from laboratory to clinic. Artificial intelligence (AI)-optimized formulations represent a paradigm shift, employing machine learning models trained on vast datasets of polymer interactions, patient genomics, and clinical outcomes to predict ideal compositions for individualized wound dressings or scaffolds that adjust degradation rates and drug release profiles autonomously. Novel incorporations like clustered regularly interspaced short palindromic repeats (CRISPR)-Cas9 payloads for on-demand gene editing, nanorobotic swarms for *in situ* diagnostics, and bioelectronic hybrids with conductive graphene for neuromodulation further amplify functionality, creating theranostic platforms that monitor disease progression while delivering therapy.

## 4 Conclusion

CS–cellulose hydrogels have demonstrated high potential as next-generation stimuli-responsive biomaterials for a wide range of biomedical applications. By synergistically integrating the intrinsic bioactivity of CS with the structural strength and hydrophilicity of cellulose, these hybrid systems have enabled the development of multifunctional platforms capable of controlled drug delivery, tissue regeneration, and targeted therapeutic interventions. Recent advances have further highlighted their adaptability through chemical modifications, crosslinking strategies, and incorporation of functional nanomaterials, resulting in enhanced mechanical properties, tunable degradation profiles, and precise responsiveness to physiological stimuli. Despite these promising developments, several critical challenges must be addressed to facilitate clinical translation. Issues related to long-term biocompatibility, immune responses, large-scale reproducibility, and the fate of degradation byproducts remain insufficiently understood. In addition, achieving precise spatiotemporal control over multi-stimuli responsiveness and ensuring consistent performance under complex *in vivo* conditions are ongoing challenges that require further investigation. Future research should focus on the rational design of smart, multifunctional hydrogel systems with improved predictability and safety profiles, supported by comprehensive *in vivo* and clinical studies. The integration of emerging technologies, such as biofabrication, artificial intelligence-assisted material design, and advanced nanocomposite engineering, is expected to further accelerate progress in this field. Therefore, CS–cellulose hydrogels represent a highly promising class of biomaterials that are poised to play a pivotal role in the advancement of precision medicine and next-generation therapeutic strategies.

## Author contributions

Nesa Rafati: writing – review & editing; Atefeh Zarepour: writing – review & editing; Arezoo Khosravi: supervision, visualization, writing – review & editing; Siavash Iravani: supervision, conceptualization, writing – review & editing; Ali Zarrabi: supervision, writing – review & editing. All authors reviewed the manuscript.

## Conflicts of interest

Author(s) declare no conflicts of interest.

## Data Availability

No primary research results, software or code have been included, and no new data were generated or analyzed as part of this review.
